# The complete chloroplast genome of *Isatis cappadocica* Desv. (Brassicaceae)

**DOI:** 10.1080/23802359.2022.2074802

**Published:** 2022-05-12

**Authors:** Zhen-Xi Fang, Xiao-Jing Qin, Qing Li

**Affiliations:** a Research and Development Center of Chinese Medicine Resources and Biotechnology, Institute of Chinese Materia Medica, Shanghai University of Traditional Chinese Medicine, Shanghai, China; b Department of Pharmacy, Second Affiliated Hospital of Naval Medical University, Shanghai, China

**Keywords:** *Isatis cappadocica* Desv., Brassicaceae, chloroplast genome, phylogeny

## Abstract

*Isatis cappadocica* Desv. is a vigorous perennial rosette plant and it can grow in highly arsenic-contaminated areas. In this study, the complete chloroplast genome of *I. cappadocica* was assembled and annotated. The total length of this genome is 153,800 bp and the GC content is 36.48%. It has a typical four-part structure: a pair of inverted repeat sequences (26,270 bp each), a small single-copy region (17,715 bp), and a large single-copy region (83,545 bp). The annotation results show that it contains 132 genes. The phylogenetic analysis of *I. cappadocica* and other 18 representative plants indicates that *I. cappadocica* is closely related to *Isatis tinctoria*.

*Isatis* L. (Brassicaceae) contains about 30 species. It is distributed in Central Europe, the Mediterranean region, and West and Central Asia. The leaves and roots of some species in this genus are used for medicine or dye (Editorial Committee of the Flora of China 1987). *Isatis cappadocica* Desv. 1815 is a member of this genus. It is a vigorous perennial rosette plant, which has the characteristics of fast growth and high biomass (Karimi et al. 2010). This plant possesses antibacterial, antioxidant, tyrosinase-inhibition and cytotoxicity activities and can be used as a natural resource for food, cosmetic, and pharmaceutical industries (Güner et al. 2019). Furthermore, it can grow in highly arsenic-contaminated areas and has the capability to hyper accumulate arsenic (Karimi et al. 2013; Souri et al. 2017). However, studies of *I. cappadocica*’s chloroplast genome are lacking. In this study, we sequenced the complete chloroplast genome of this plant and performed a phylogenetic analysis of *I. cappadocica* with 18 representative plants.

The seeds of *I. cappadocica* (origin from Turkey) were obtained from the medicinal botanical garden of Naval Medical University, Shanghai, China, and they were planted at Shanghai University of Traditional Chinese Medicine (N31°11′36.20′′, E121°35′50.96′′, Shanghai, China). A specimen was deposited in the herbarium of Shanghai University of Traditional Chinese Medicine (https://www.shutcm.edu.cn, Wansheng Chen, chenwansheng@smmu.edu.cn) under the voucher number IsCAP001 and it was identified by Prof. Wansheng Chen.

The young leaves of *I. cappadocica* were collected in accordance with the guidelines provided by the Shanghai University of Traditional Chinese Medicine and granted by the National Natural Science Foundation of China for total genomic DNA extraction with the DNeasy Plant Mini kit (QIAGEN Bio-Tec). DNA extracts were fragmented into 150 bp with a Covaris^®^ M220 focused-ultrasonicator™ (Covaris, Woburn, MA, USA) and sequencing was conducted on an Illumina HiSeq X Ten instrument at Novogene Biotech Co., Ltd. (Beijing, China). Clean data were *de novo* assembled into a complete chloroplast genome by GetOrganelle (Jin et al. 2020), and the finished chloroplast genome was annotated with CPGAVAS2 (http://47.96.249.172:16019/analyzer/annotate). Finally, the annotated chloroplast genome of *I. cappadocica* was submitted to GenBank with the accession number OL404951.

The sequence analysis shows that the whole chloroplast genome of *I. cappadocica* is 153,800 bp in size and its GC content is 36.48%. The chloroplast genome has a typical four-part structure, with two inverted repeat regions (IR) of 26,270 bp each (42.33% GC contents), separated by a small single-copy region (SSC) of 17,715 bp (29.68% GC contents) and a large single-copy region (LSC) of 83,545 bp (34.25% GC contents). The complete chloroplast genome of *I. cappadocica* contains 132 total genes, including 87 protein-coding genes, 8 rRNA genes, and 37 tRNA genes.

To investigate the phylogenetic relationship of *I. cappadocica*, the molecular phylogenetic tree was constructed based on the common genes of the complete chloroplast genome of *I. cappadocica* and other 18 plants, which were retrieved from GenBank. The chloroplast genomes were aligned by PhyloSuite v1.2.2 (Zhang et al. 2020, https://github.com/dongzhang0725/PhyloSuite/releases) and the maximum-likelihood (ML) analysis was conducted with a bootstrap of 1000 repetitions based on the GTR + F + I + G4 nucleotide substitution model (Nguyen et al. 2015). The phylogenetic analysis indicated that *I. cappadocica* has a close relationship with *Isatis tinctoria* (Figure 1), a famous indigo-producing plant.

**Figure 1.**
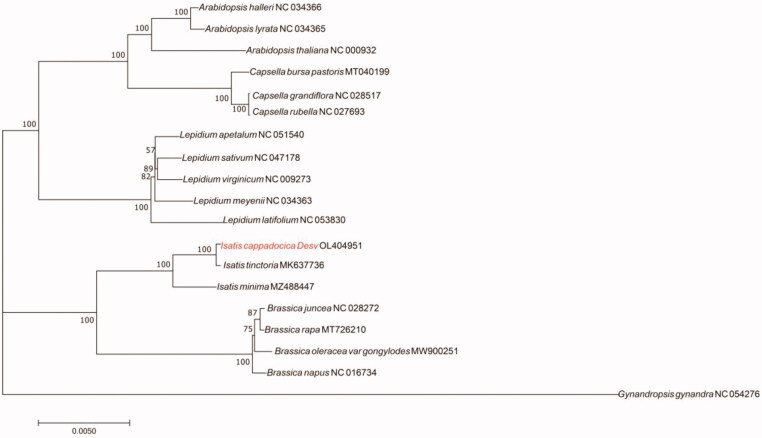
Maximum-likelihood (ML) phylogenetic tree based on the complete chloroplast genome sequences of *I. cappadocica* and 18 representative species. Red font is the species studied in this paper and bootstrap support values are shown on the nodes.

## Data Availability

The chloroplast genome sequence data that support the findings of this study are openly available in GenBank of NCBI (https://www.ncbi.nlm.nih.gov/) under the accession No. OL404951. The associated raw sequencing data have been deposited into the National Genomics Data Center, China National Center for Bioinformation/Beijing Institute of Genomics, Chinese Academy of Sciences, under the Genome Sequence Archive number CRA006127 with the BioProject number PRJCA008257 and the BioSample number SAMC606263 (https://ngdc.cncb.ac.cn/).
